# Improving the intensive care experience from the perspectives of different stakeholders

**DOI:** 10.1186/s13054-022-04094-x

**Published:** 2022-07-18

**Authors:** Jos M. Latour, Nancy Kentish-Barnes, Theresa Jacques, Marc Wysocki, Elie Azoulay, Victoria Metaxa

**Affiliations:** 1 School of Nursing and Midwifery, Faculty of Health, University of Plymouth, Plymouth, UK; 2 School of Nursing, Midwifery and Paramedicine, Faculty of Health Sciences, Curtin University, Perth, Australia; 3 Famirea Study Group, Medical Intensive Care Unit, Assistance Publique-Hȏpitaux de Paris, Saint Louis University Hospital, Paris, France; 4 Department of Intensive Care, St George Hospital, Sydney, Australia; 5 St George and Sutherland Clinical School, University of New South Wales, Sydney, Australia; 6 Life Care and Digital Solutions, GE Healthcare, Wauwatosa, WI 53226 USA; 7 Department of Critical Care, King’s College Hospital NHS Foundation Trust, London, UK

**Keywords:** Intensive care unit, Patients, Family, Healthcare professionals, Experiences, Perceptions, Comfort, Quality of health care, Industry

## Abstract

The intensive care unit (ICU) is a complex environment where patients, family members and healthcare professionals have their own personal experiences. Improving ICU experiences necessitates the involvement of all stakeholders. This holistic approach will invariably improve the care of ICU survivors, increase family satisfaction and staff wellbeing, and contribute to dignified end-of-life care. Inclusive and transparent participation of the industry can be a significant addition to develop tools and strategies for delivering this holistic care. We present a report, which follows a round table on ICU experience at the annual congress of the European Society of Intensive Care Medicine. The aim is to discuss the current evidence on patient, family and healthcare professional experience in ICU is provided, together with the panel’s suggestions on potential improvements. Combined with industry, the perspectives of all stakeholders suggest that ongoing improvement of ICU experience is warranted.

## Introduction

Critical illness impacts patient and relatives. Evidence suggests that prolonged intensive care unit (ICU) stay is associated with physical, mental, cognitive and psychological sequelae for ICU survivors, which can persist long after ICU discharge (Post-ICU Syndrome). Decision-making during ICU stay is often shared with patient’s relatives, which can increase the inherent anxiety and depression from having a loved-one in the ICU [1]. Furthermore, the ICU environment is an emotional place for healthcare professionals, who experience challenging situations that provoke conflicting emotions such as isolation, sadness, anger, shame, love, and happiness [2].

Structured interventions and approaches aimed at improving patient, family and healthcare experiences have recently been the focus of research in the ICU [3–5]. We present an overview of the discussion raised by a panel of experts, who participated in a GE Healthcare-sponsored symposium held during the LIVES2021 congress of the European Society of Intensive Care Medicine (ESICM). This was a multi-national and multi-disciplinary symposium with presentations from colleagues with extensive experience in ICU. We decided to add representation from the sponsoring company, recognising the importance of technology in creating an optimum ICU environment. The aim of this report is to discuss and present expert suggestions that may improve the ICU experience of patients, their relatives, and healthcare professionals, including the perspectives of industry.

## The patient perspective

Individual aspects of patient experience, such as quality of sleep, pain and sedation, are measured during ICU admission to guide and assess our interventions. The ICU survivors recall their experience to varying degrees and their recollection may be factual or illusory [6]. Measuring and understanding recalled patient discomfort has the potential to provide a global measure of patient ICU experience.

### Measuring recalled discomfort

Van de Leur et al. demonstrated a link between patient’s factual recall of ICU events and the recollection of discomfort experienced during an ICU stay [7]. Focusing on recalled discomfort is important because it is associated with post-ICU syndromes, such as sleep disturbance, anxiety, mood disorders and Post-Traumatic Stress Disorder (PTSD).

The validated IPREA (*Inconforts des Patients de REAnimation*) questionnaire measures perceived or recalled discomfort from an ICU episode and can be used irrespective of the diagnosis, the disease or the organ support the patient receives. The ICU survivors are asked at ICU discharge about possible causes of discomfort, using an 18-item questionnaire, and rate the severity of each cause. The questionnaire has been translated into English [8]. The IPREA studies show that sleep deprivation, discomfort due to lines and tubes, pain, and thirst are the highest scored items on the discomfort scale [9], with ICU experiences of discomfort being similar across countries and cultures.

### Improving patient experience

**Fig. 1:**
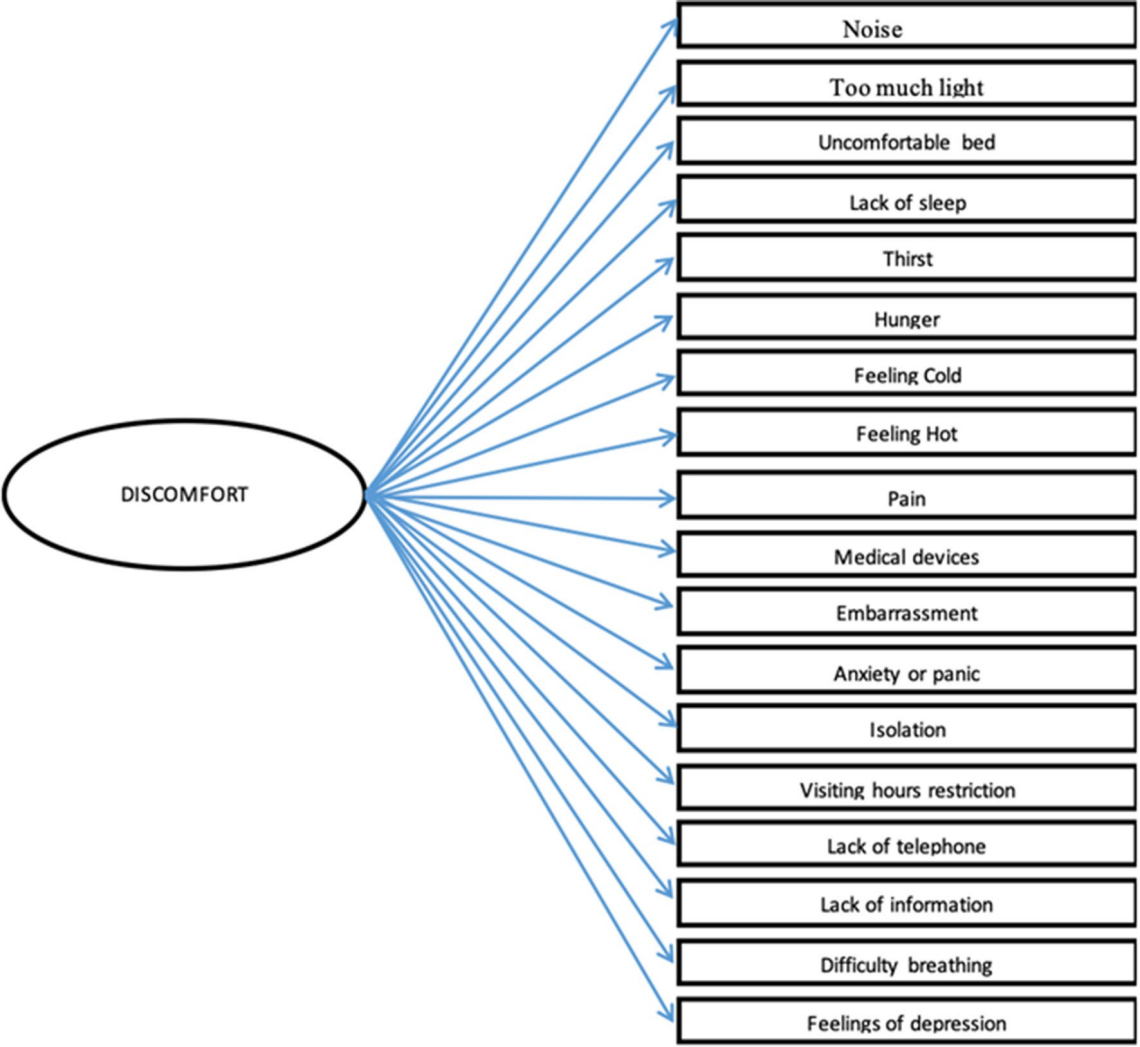
ICU factors related to discomfort

Consideration of the 18 domains of discomfort in Fig. 1 (adapted from Kalfon et al. [10]) should be incorporated in ICU daily practice. By understanding patient experiences and components of their discomfort clinicians can modify the ICU environment, the care provided and the communication with patients. Environmental factors in ICU design that should be considered include noise reduction, provision of natural light, presence of a clock, telephone and TV, as well as maintaining privacy. Aspects of ICU care such as visiting hours, communication of information, mouth and airway care, pain and sedation are paramount in delivering high quality and safe care to ICU patients.

The incidence of PTSD in ICU survivors is approximately 20%. There have been mixed successes in studies using interventions to mitigate PTSD development in the post-ICU phase. The POPPI study, a nurse-led preventative psychological intervention among ICU survivors, did not demonstrate significant reduction in PTSD symptoms at 6 months [11]. In contrast, the IPREA AQVAR group published a tailored multi-component programme, which used comfort champions and local strategies and showed a significant reduction in overall discomfort and a decrease in PTSD at 12 months post-ICU discharge [12]. Another recent study reported reduction in PTSD symptoms using a virtual reality programme for ICU discharged patients [13].

Exploring discomfort post-ICU discharge can provide insights into patient ICU experiences and the impact of quality of care. The incidence or severity of post-ICU syndromes may be reduced by addressing various aspects of discomfort, but more research is warranted. Suggestions to reduce discomfort among ICU patients are presented in Table 1. The COVID-19 pandemic has highlighted even more the importance of assessing ICU patient experiences and the long-term impact of critical illness and ICU interventions.

**Table 1:** The patient experience and suggestions for improvements

Assessing the ICU patient perspective to improve experience
1. Explore aspects of the patient experience in the broadest terms
2. Consider patient factors, environment factors, care & intervention factors
3. Patient responses preferred to surrogate
4. Assess the patient experience early in the post ICU phase for recollection of the ICU experiences after excluding delirium
5. Assess the patient experience later for adverse sequelae (anxiety, depression, post-traumatic stress disorder)
6. Link the aspects of the patient experience back to the quality of ICU care

## The family perspective

Family-centred care is defined as an approach to healthcare that is respectful of and responsive to individual families’ needs and values, and in which partnership and collaboration are key concepts [4, 14]. Research has contributed to develop family-centred care by helping clinicians to better understand and improve family members’ experience.

### Humanizing the ICU

Debates over closed versus open visiting policies have been numerous, with significant variations in practice between and within countries [15, 16]. Unfortunately, the COVID-19 pandemic generated a considerable setback, as ICUs felt compelled to restrict visiting. Importantly, open visiting policies are associated with decreased anxiety and better understanding of information [17]. In the technical ICU environment, qualitative research has provided dimensions of humanization important to family members, such as personalization (vs. objectification), agency (vs. passivity), togetherness (vs. isolation) and sense-making (vs. loss of meaning) [18]. Moreover, families in the ICU are sensitive to clinicians’ empathy and to reciprocal relationships [19].

### Families’ psychological burden

Family members are extremely vulnerable during the patient’s ICU stay. They only understand approximately half of the medical information given to them by the ICU team [20], generating difficulties to adapt and manage hope. Families also remain vulnerable after the patient’s discharge or death. Three months post-ICU discharge, up to 70% suffer from symptoms of anxiety, 35% from symptoms of depression [21], and up to one third suffer from PTSD-related symptoms [22].

### Communication

Communication is at the heart of the family’s experience. It consists of verbal communication (words) and non-verbal communication (body language), the latter determining the quality of the speakers’ message and its ability to be received [23]. In highly emotional situations, such as being in ICU, family members are extremely sensitive to non-verbal communication. The quality of overall communication impacts on relatives’ well-being: unsatisfactory communication is associated with higher risk of developing PTSD related symptoms [22] and in bereaved relatives, it is associated with increased risk of developing complicated grief at 6 and 12 months after the patient’s death [2].

### Improving family experience

Most randomized controlled trials aiming to improve families’ wellbeing have focused on improving communication between ICU clinicians and relatives. The Family End-of-Life Conference, a meeting between the patient’s clinicians and the family, encourages clinicians to **V**alue family statements, **A**cknowledge family emotions, **L**isten to the family, **U**nderstand the patient as person and **E**licit questions from families [3]. In a French trial, this pro-active communication strategy was associated with a decreased risk of developing anxiety, depression and PTSD related symptoms three months after the patient’s death [24]. Including a nurse facilitator in the family conferences was associated with a decreased risk of developing depression symptoms in family members 6 months after the patient’s ICU discharge or death [25]. Furthermore, a three-step support strategy for relatives of patients dying after a decision to withdraw treatment, including a family conference before the patient’s death, a room visit during dying and death, and a meeting after the patient’s death, was associated with a decreased risk of developing prolonged grief, as well as anxiety, depression and PTSD related symptoms 6 months after the patient’s death [26]. More research is needed to evaluate the developed strategies as some interventions have proven to be deleterious [27, 28]. Suggestions to improve the family experiences are presented in Table 2.

**Table 2:** The family experience and suggestions for improvement

Discovering a “new world”	Open/flexible visiting policies
Coping with medical jargon	Use family leaflets and digital materials
Difficulties in understanding information (inadequate timing, language barriers, contradictory information)	Improve communication skills
Reporting impersonal information	Adapt words, use reformulation (tell-ask-tell), and when necessary, an interpreter
Psychological distress at any time during the ICU stay:	Allow more time for information
Acute stress	Develop empathetic communication, verbal and nonverbal communication
Anxiety	Encourage
Depression	Personalization
Peritraumatic dissociation	Agency
Psychological distress in the months that follow ICU discharge:	Togetherness
Anxiety	Sense-making
Depression	Regular interprofessional family meetings, including the nurse
PTSD	Family End of Life conference (VALUE acronym)
Complicated grief	Nurse communication facilitator
Other difficulties	Physician and nurse support strategy before, during and after the patient’s death
Sleep disorders and sleepiness	Available psychologist
Managing family and work life	Available social worker
Financial stress	
Transfer anxiety	

## The healthcare professionals’ perspective

The COVID-19 pandemic generated a new dimension on the experiences of ICU professionals. Survey studies have indicated the increase physical and psychological burden of ICU staff while caring for COVID patients [29–31]. Qualitative studies generated a deeper understanding of the impact [32, 33], which can be summarised as the ‘emotional impact affecting the personal self’, the ‘professional fellowship among colleagues’ and the ‘recognition and support from the outside’.

### Emotional comfort

The experiences of ICU healthcare professionals have mainly been studied by qualitative research methods [5, 34]. In these studies, a range of emotions have been identified, with one of the six reported themes being that of emotional impact [35]. Within this theme, ICU nurses addressed empathy as an important skill to develop, whereas for ICU doctors, the overarching themes were the risk and benefits of empathy, the spectrum of connection and distance from patients/families, and the facilitators and barriers to empathy development [36]. A scoping review indicated that empathy among intensivists is not a dichotomous phenomenon and that a deeper understanding is needed to create a supportive environment where ICU professionals feel safe to demonstrate their empathy to patients and relatives [36].

### Complexity of decision-making

The complexity of ICU patients and their pathway to recovery or death influences the performance of ICU staff and impact on their mental health. This complexity does not only relate to caring for certain patient groups but also to participation in decision-making. The involvement in decisions relating to treatment withdrawal or organ donation has been challenging for many ICU professionals [37–39]. The low research priority given to delirium care has caused frustration to ICU nurses, due to the resulting lack of confidence in assessing delirium.^15^ Most studies conclude that continuous specialist education is required to provide high quality-of-care to the increasingly complex ICU patient.

### Improving healthcare professional experience

Improving the ICU experience of healthcare professionals is necessary in order to maintain safe ICU environment, high quality ICU staffing and a sustainable workforce. It is essential for the formation of a positive ICU climate, which will help healthcare professionals cope with the most complex needs of ICU patients and relatives, and provide high quality of care [40, 41]. Staff empathy skills can be taught, as demonstrated by a 5-day course on empathy education, including simulation training, which significantly increased the empathy levels of student nurses [42]. Further suggestions to support the health and well-being of ICU health professionals are presented in Table 3.

**Table 3:** Health and well-being support for ICU professionals

*Interventions individual level*
Provide a buddy-system to support colleagues
Provide confidential counselling
Support equality diversion and inclusion by peer-to-peer learning system
Organise daily staff huddles to support teamwork
Provide and ensure regular breaks
Provide the basics in staff room—facilities, food trolley, drinks
Create a system to follow-up on sick leave
*Interventions team level*
Promote mutual respect within the multi-professional team
Allow staff for self-reflection and empowering to lead change
Organise regular ICU staff discussions facilitated by a psychologist or ethicist
Create a culture of not avoiding end-of-life discussion
Support interprofessional shared decision making
Employ end-of-shift debrief sessions
Continuous training sessions on communication, feedback, mindfulness, mediation
*Interventions organisational level*
Create a safe environment for ICU staff to feel comfortable
Monitor the ‘need for recovery’ between shifts and act on the results
Organise thanks and award/reward systems
*Interventions external stakeholders level*
Create an information and inclusion system for hospital staff connected to the ICU
Initiate a (digital) support and information system for family members of ICU staff

## The industry perspective

Professional organizations are describing the ICU as *‘very daunting place… equipped with many devices to monitor the patients… sophisticated machines and screens... alarms… with the devices connected to a central station…’* [43].

### Medical devices and impact on comfort

Medical devices, such as ventilators, renal replacement equipment, infusion pumps and extracorporeal membrane oxygenators have the potential to influence patients’, families’ and healthcare professionals’ ICU experience. In Fig. 1 it is obvious that discomfort is often generated by medical equipment, such as alarms inducing excess of noise or lines, tubes and cables constraining the patient. Noise is a common source of patient discomfort and may have negative impact on the visiting family and healthcare professionals [10]. By mapping the various sources of noise in ICU, Darbyshire et al. found that a significant proportion originated from equipment alarms in extremely limited areas, very close to patients’ ears [44].

### Improving by digital transformation

**Fig. 2:**
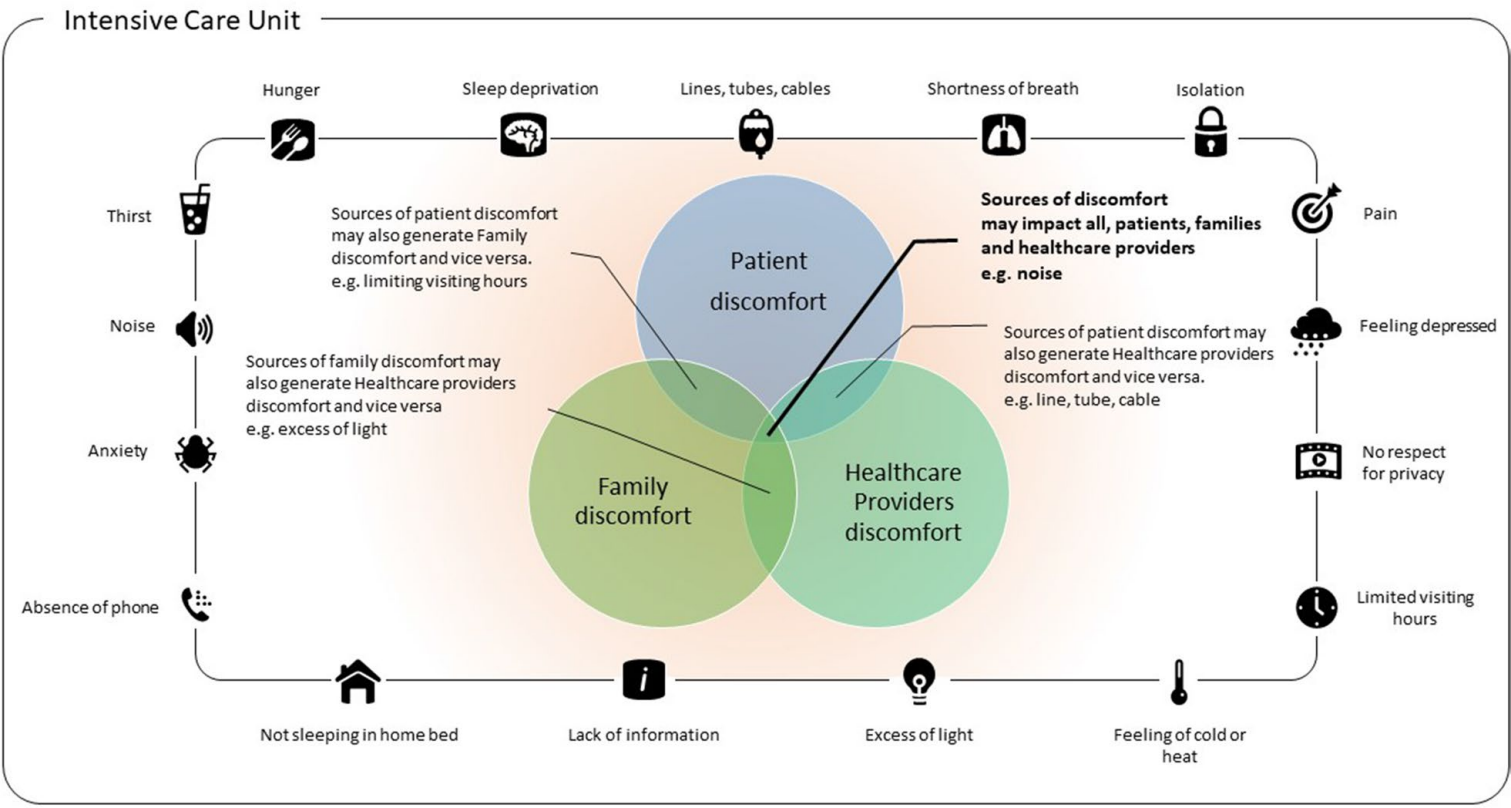
Conceptual framework for an holistic approach of discomfort in the ICU.

The contribution of industry can have a positive impact on the entire ICU ecosystem (Fig. 2). By digital adjustment and automatization, the unavoidable clerical burden needed for resource allocation and documentation, can be alleviated, allowing staff to dedicate their time to spending clinical time with patients and the families. In an 18-bed academic medical-surgical ICU, Bosman et al. reported a 30% reduction in documentation time by using a clinical information system at the bedside; time, which was completely re-allocated to patient care [45]. The digital transformation of ICU helps reduce not only the documentation burden but also improves patient comfort and family engagement and communication. Dashboards displaying discomfort scores may act as reminders and influence the provided care, enhancing ICU experience. A dedicated ICU clinical information system may also general reminders to alert ICU staff that a communication with the family is needed and thus preventing potential conflict.

If patient’s discomfort is relatively well documented (Baumstarck 2014), family discomfort and healthcare provider discomfort need to be further investigated. The concept is assuming that some of the source of discomfort are unavoidably shared by all the participants (patient, family, healthcare providers). Improving ICU experience by reducing discomfort may be best achieved by considering the entire ICU ecosystem, including peoples (patient, family, healthcare providers), various workflow and process and the surrounding medical equipment and devices.

A redesign of the ICU environment to move alarm sounds away from the bedside may significantly reduce noise-related discomfort. Improving the operational value and the usability of alarm signals, without being unnecessarily distracting or disturbing, is also the goal of recently updated safety standards (ISO 60601-1-8) which need to be followed by manufacturing companies. Sophisticated stand alone or embedded alarm management solutions have been developed not only to reduced noise-related discomfort but also to avoid family anxiety and caregivers’ annoyance and alarm fatigue [46]. Collaboration in equipment design and digital solutions between clinicians, patients and industry is part of the solution for stakeholder experience in ICU.

## Discussion

Critically ill patients experience various discomforts during their ICU stay, that may be related to the environment (noise, light, temperature, etc.), some aspects of care organisation (continuous light, limited visiting hours, lack of privacy, etc.), and also specific ICU therapeutics (mechanical invasive and non-invasive ventilation renal replacement therapy, or painful procedures). This conference paper has focused on interventions that may enhance ICU experience not only for patients but also for families and critical care staff. The daily assessment and recognition of potential patient discomfort in ICU will ensure greater insight into their experience and improve the quality of the offered care. Improving communication both at an individual but also at a collective level has been highlighted as the most important intervention for improving family experience, by making family-centred care a quality standard. Revisiting ICU staffing models and training of nurses and doctors on empathy and communication skills are important in order to create a positive ICU climate with a sustainable workforce. The transparent involvement and collaboration of industry in developing tools and technologies that are aimed at humanising the ICU environment is increasingly recognised as an important part of the equation.

## Data Availability

Not applicable**.**
